# How the Metaverse Is Shaping the Future of Healthcare Communication: A Tool for Enhancement or a Barrier to Effective Interaction?

**DOI:** 10.7759/cureus.80742

**Published:** 2025-03-17

**Authors:** Alexandru Burlacu, Crischentian Brinza, Nicolae Nichifor Horia

**Affiliations:** 1 Cardiology, Faculty of Medicine, Grigore T. Popa University of Medicine and Pharmacy, Iași, ROU; 2 Cardiology, Prof. Dr. George I.M. Georgescu Institute of Cardiovascular Diseases, Iași, ROU; 3 Orthodox Theology, Faculty of Orthodox Theology, Alexandru Ioan Cuza University, Iași, ROU

**Keywords:** augmented reality, healthcare, medical education, metaverse, technology, virtual reality

## Abstract

The metaverse is emerging as a transformative force in healthcare communication, integrating virtual reality (VR), augmented reality (AR), artificial intelligence (AI), and extended reality to enhance doctor-patient interactions, interprofessional collaboration, medical education, and surgical planning. By providing immersive, interactive, and data-driven environments, the metaverse could facilitate real-time consultations, remote surgical assistance, and simulation-based training, overcoming traditional geographical and logistical barriers. Despite these advancements, skepticism persists regarding the metaverse’s true benefit in fostering meaningful human interaction. Some critics argue that virtual interfaces risk alienating communication, eroding the depth of doctor-patient relationships rather than strengthening them. The concern remains that digital mediation might replace rather than enhance human presence, diminishing the nuances of empathy and trust inherent in face-to-face interactions. Economic constraints, technological disparities, and the potential reduction in direct human interaction can complicate widespread adoption. Some perspectives suggest that, if strategically implemented, the metaverse could foster a more human, authentic, and profound doctor-patient relationship by reducing administrative burdens and allowing physicians to focus more on patient care. While the metaverse holds promise for revolutionizing digital healthcare, its long-term success depends on responsible implementation, equitable access, and strategic integration into existing healthcare frameworks. In this paper, we aim to critically evaluate both sides of this debate, synthesizing existing evidence to clarify the role of the metaverse in future healthcare communication.

## Introduction and background

The metaverse represents a transformative digital paradigm, merging virtual and physical realities through immersive technologies such as virtual reality (VR), augmented reality (AR), artificial intelligence (AI), blockchain, and digital twins. VR and AR constitute immersive technologies that either create entirely simulated environments or overlay digital information onto real-world scenarios, significantly enriching user interactions and experiences. Blockchain is a decentralized digital ledger technology that securely records transactions across multiple participants, ensuring transparency and data integrity. A digital twin represents a virtual model replicating a physical entity, enabling accurate simulations and analyses that help optimize real-world performance and decision-making. While initially conceptualized for entertainment and social interaction, the metaverse expansion into healthcare has opened new avenues for medical education, telemedicine, surgical planning, and patient rehabilitation [1, 2].

In the medical domain, the metaverse facilitates real-time, interactive communication between healthcare professionals, patients, and medical students, overcoming geographical limitations and enhancing accessibility to high-quality care​ [3]. Integrating AI-driven analytics and extended reality in virtual environments enables more personalized healthcare delivery, advanced diagnostic simulations, and enhanced procedural training [4]. An example of these benefits playing out in real-time came with the COVID-19 pandemic, where the adoption of digital health interventions demonstrated the feasibility and benefits of remote consultations, virtual surgical training, and AI-assisted decision-making [5].

Despite its potential, the implementation of metaverse technologies in medicine remains in its nascent stages. Significant challenges persist, including high costs, concerns about data security, ethical considerations, and disparities in technology access​ [3]. As the healthcare industry navigates these complexities, it is imperative to explore the capabilities, benefits, and limitations of integrating metaverse-based solutions in clinical and educational settings​ [5].

Integrating immersive technologies in healthcare is not merely an enhancement but a potential revolution in how medical professionals interact with patients, collaborate with colleagues, and refine their clinical skills. Extended reality platforms offer a dynamic space where AI-based decision support systems and virtualized patient simulations can replicate real-world medical environments, providing clinicians with more accurate diagnostic tools and hands-on learning experiences [2, 6]. These advancements improve patient engagement and foster a more interactive and adaptive medical education system, where knowledge transfer, skill acquisition, and competency assessment can occur in a risk-free, controlled environment​ [1].

This article aims to provide an overview of the metaverse's communication dynamics, educational impact, and clinical medical applications while addressing existing challenges and prospects. This review will synthesize current evidence and expert insights and examine how the metaverse reshapes healthcare delivery, medical education, and patient-centered care, offering new frontiers for digital medicine.

## Review

Materials and methods

To identify relevant literature, a comprehensive search was conducted in PubMed, Web of Science, Cochrane Library, Embase, and Scopus for studies published up to February 1, 2025. Several keywords were used in the search process, including "metaverse," "healthcare," "virtual reality," "augmented reality," "telemedicine," "surgical simulation," "digital healthcare," "artificial intelligence," "remote medical collaboration," and "extended reality." The search was not restricted by publication language.

Studies were included if they specifically discussed the applications of the metaverse in medicine, such as its use in virtual patient care, surgical planning, medical education, and interdisciplinary collaboration. Eligible articles included original research, systematic reviews, and meta-analyses published in peer-reviewed journals. Exclusion criteria included studies that primarily focused on non-medical applications of the metaverse, theoretical discussions without practical implementation in healthcare, and opinion pieces, editorials, case reports, or conference abstracts that lacked empirical data.

The study selection process involved an independent review of article titles and abstracts by two researchers, who excluded studies that did not meet the predefined eligibility criteria. Then, the full text of the remaining articles was assessed, and disagreements regarding inclusion were resolved through discussion or consultation with a third reviewer.

Data from the selected studies were extracted and analyzed to identify key themes and trends in implementing metaverse-driven healthcare solutions. The extracted information included the specific medical application, technologies, reported benefits and limitations, and any ethical and security concerns related to digital patient data sharing and interoperability. A qualitative synthesis was performed to provide a comprehensive overview of how the metaverse transforms various aspects of healthcare, from medical education and training to clinical decision-making and remote collaboration.

**Figure 1:**
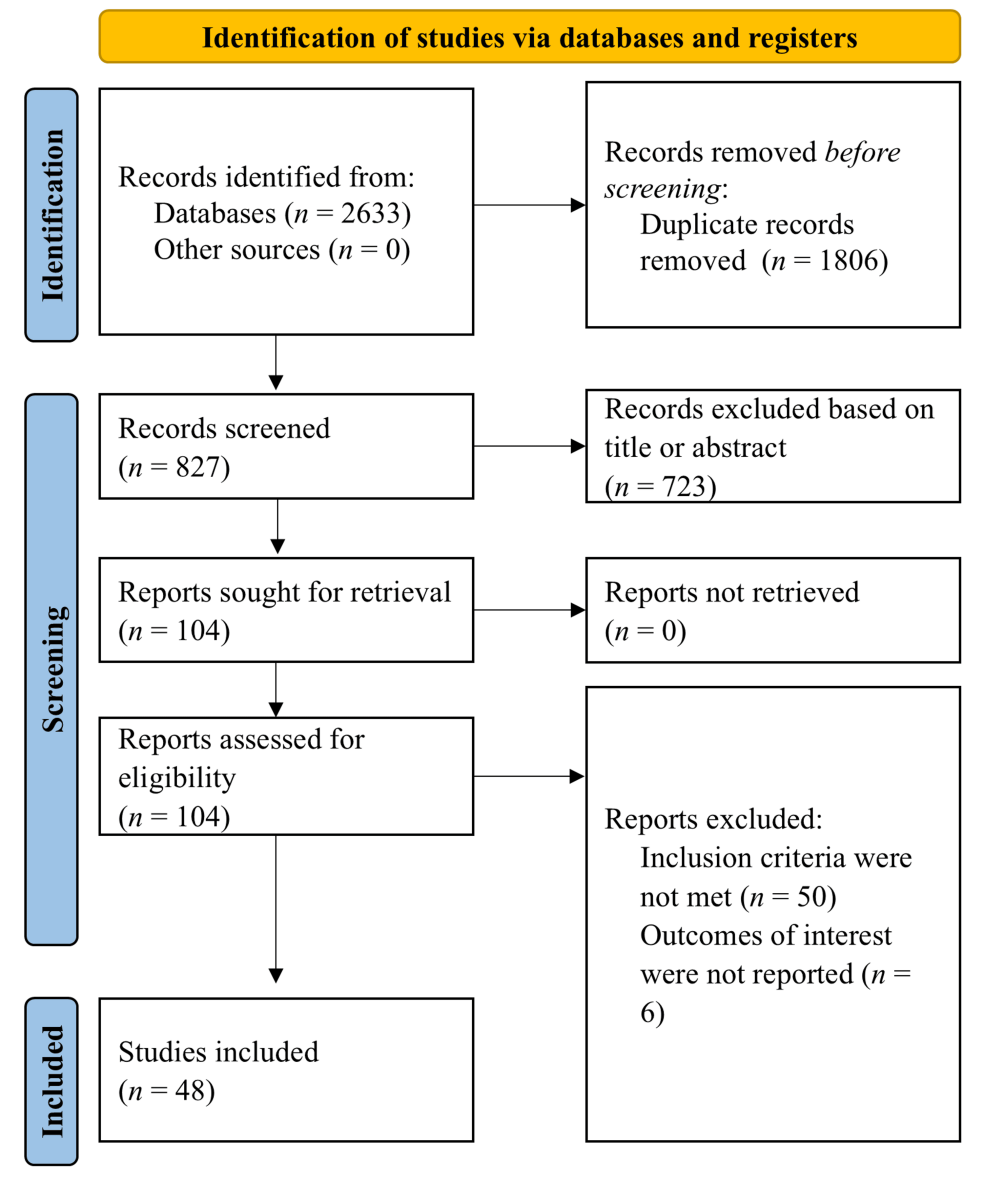
Flow chart of selecting studies in the present analysis.

A total of 2,633 records were identified through database searching. After removing duplicates, 827 records remained for title and abstract screening. Based on this screening, 723 records were excluded, leaving 104 reports for full-text retrieval and eligibility assessment. After a detailed full-text review, 56 reports were excluded, and 48 studies were included in the qualitative synthesis (Figure 1).

Metaverse as a communication platform in medicine

Enhancing Doctor-Patient Interactions

The metaverse offers a novel medium for doctor-patient interactions by enabling immersive virtual consultations. Through interactive 3D environments, patients can engage in real-time discussions with healthcare providers, facilitating a more personalized and engaging experience than traditional telemedicine platforms [7-9]. This approach can improve patient satisfaction and adherence to treatment plans by fostering a sense of presence and empathy in virtual settings. Furthermore, integrating AI within these platforms can assist in real-time translation services, making healthcare more accessible to non-native speakers and individuals with hearing impairments [7,8].

Transforming Interprofessional Communication

The metaverse also serves as a dynamic space for interprofessional communication among healthcare teams. VR platforms enable the simulation of clinical scenarios where medical professionals can collaborate, practice, and refine their skills in a risk-free environment [10]. For instance, VR-based simulations have been utilized to enhance teamwork and communication in emergency medicine, allowing teams to practice protocols and improve response times without the constraints of physical simulation centers. Additionally, metaverse platforms can host virtual conferences and workshops, enabling continuous professional development and knowledge exchange across geographical boundaries [9,11,12]. ​

Leveraging the metaverse's immersive and interactive capabilities can significantly enhance doctor-patient and interprofessional communications, leading to improved healthcare outcomes and more efficient medical practices [13,14].

Metaverse in Virtual Healthcare Collaboration

Metaverse-based virtual hospitals enable multidisciplinary teams to conduct comprehensive patient evaluations and treatment planning without the constraints of physical location. A study demonstrated the feasibility of conducting complex multidisciplinary team meetings using an immersive VR metaverse platform on commercially available headsets. Participants reported effective data visualization and clinical decision-making, highlighting the potential of the metaverse to enhance collaborative medical practices. Also, virtual hospitals implemented in several countries could offer multidisciplinary team meetings in virtual immersive spaces, enabling remote specialists to collaboratively plan complex procedures. Additionally, platforms such as XRHealth deliver virtual therapy and surgical training, respectively, highlighting practical applications that translate immersive technologies from theory into clinical practice. These platforms demonstrate clear benefits, including reduced logistical barriers, standardized educational scenarios, and enhanced remote patient monitoring [15].

Real-time surgical collaboration is another significant advancement facilitated by the metaverse. Through shared virtual environments, surgeons from different locations can participate simultaneously in the same procedure, allowing immediate consultation and assistance. This capability enhances surgical outcomes and is a valuable educational tool for training surgeons. A mini-review with video content has showcased the safety and efficiency of metaverse technology in surgical settings, indicating its potential to transform traditional surgical practices [16].

However, integrating the metaverse into healthcare communication raises ethical and privacy concerns regarding digital patient data sharing and interoperability. The extensive data sharing required to facilitate interoperability in the metaverse presents unique challenges in preserving patient privacy and confidentiality. A comprehensive review has highlighted the importance of addressing these ethical hazards to ensure responsible health data governance within the metaverse [17].

Metaverse in medical education and training

Integrating metaverse technologies revolutionizes medical education by offering immersive, interactive, and context-rich learning experiences. By leveraging VR, AR, and mixed reality (MR), educators can create simulation-based training environments that enhance skill acquisition and competency development among medical professionals [10,18-22]. Studies investigating the use of metaverse technologies in medical education are summarized in Table 1.

**Table 1:** Studies on metaverse technologies in medical education.

First author	Year	Key findings
Garcia-Robles et al. [20]	2024	Knowledge performance using extended reality devices was increased compared to textbooks and atlases (SMD = 0.32; 95% CI = 0.10 to 0.54) and didactic lectures (SMD = 1.00; 95% CI = 0.57 to 1.42). Additionally, 80% of students reported these devices as helpful in learning anatomy.
Kyaw et al. [21]	2019	VR demonstrated a modest enhancement in post-intervention knowledge acquisition compared to traditional learning methods, as indicated by an SMD of 0.44 (95% CI, 0.18–0.69) based on data from 603 participants. VR enhanced cognitive skills among healthcare professionals more effectively than traditional learning methods, with an SMD of 1.12 (95% CI, 0.81–1.43).
Uruthiralingam et al. [22]	2020	A systematic review which assessed the effectiveness of AR in medical education. Most studies reported positive effects on learning outcomes, including improved knowledge retention and skill performance.
Moro et al. [23]	2021	A literature review highlights the potential benefits for educators in prioritizing assessment strategies that evaluate three-dimensional spatial understanding in physiology and anatomy. While overall findings indicate that test performance does not significantly improve with either approach, virtual and augmented reality present viable alternatives to conventional health sciences and medical training educational methods.
Salimi et al. [24]	2024	A systematic review assessed the effectiveness of VR and AR in anatomy education. The results suggested that VR serves as a valuable tool for teaching anatomy in medical training. In contrast, AR did not demonstrate a significant influence on learning outcomes.
Lau et al. [10]	2025	The reviewed publications highlighted numerous advantages of metaverse technologies: the flexibility of time and location, cost-effectiveness, a high degree of experimental control, interactivity, the ability to facilitate low-risk learning environments, multiuser capabilities, data generation, and immediate feedback.

VR has emerged as a transformative tool in medical education, enabling the creation of immersive environments where students can interact with 3D anatomical models and simulate clinical scenarios. This technology facilitates experiential learning, allowing for the practice of procedures and decision-making in a risk-free setting. A scoping review highlighted the evolution of simulation technology with the emergence of metaverse applications in medical training, emphasizing the potential of VR to enhance educational outcomes [24].

AR overlays digital information in the real world, providing real-time guidance during medical procedures. This technology enhances clinical skill development by allowing trainees to visualize anatomical structures and receive interactive feedback during practice sessions. ​ In orthopedic trauma surgery training, AR-based simulations have been utilized to provide versatile, patient-specific training scenarios. These simulations are adaptable to individual trainees and reduce the risk to patients, enhancing the learning experience [25]. ​

The integration of metaverse technologies into medical education has been explored in various contexts, including acute care settings. A scoping aimed to provide a comprehensive overview of research describing metaverse use in education for emergency, critical, and acute care [10]. The review highlighted that metaverse technologies, encompassing VR, AR, and MR, offer immersive and interactive learning experiences. These technologies enable simulation-based training to enhance skill acquisition and competency development among medical professionals. The immersive nature of the metaverse allows for realistic simulations of acute care scenarios, providing learners with opportunities to practice and refine their skills in a safe and controlled environment [10].

However, the review also identified challenges in implementing metaverse technologies in medical education. These include high costs, technical obstacles, and limited resource availability. Addressing these challenges is crucial to fully harnessing the metaverse's potential to enhance medical education, particularly in high-stakes acute care settings [10].

While the metaverse offers significant advancements in medical education, concerns arise regarding its potential impact on clinical skills and diagnostic intuition among young physicians. The reliance on virtual simulations and AI-driven diagnostics may inadvertently lead to a diminished ability to perform thorough physical examinations and interpret non-verbal patient cues. Direct patient interaction provides invaluable experiential learning, fostering intuition and critical thinking that cannot be entirely replicated in a digital environment. In this sense, the metaverse could act as a Trojan horse by introducing impressive technological enhancements while subtly eroding fundamental clinical competencies [26]. A balanced integration of metaverse-based education with hands-on, patient-centered learning experiences is essential to mitigate this risk.

Overall, while metaverse technologies promise to transform medical education through immersive and interactive learning experiences, further research is needed to evaluate their effectiveness and address implementation challenges. Future studies should focus on assessing the impact of these technologies on learning outcomes and clinical practice to ensure their effective integration into medical education curricula. While current literature demonstrates that metaverse technologies enhance educational outcomes, evidence of the direct impact on clinical outcomes, such as patient satisfaction, therapeutic adherence, or symptom improvement remains relatively limited. Some studies have indeed suggested potential clinical benefits, including improved patient outcomes, reduced healthcare costs, and increased patient satisfaction. However, further large trials are required before broad clinical application can be recommended [10].

Discussion

Pros: A Tool for Enhancement

The metaverse introduces unprecedented opportunities for healthcare communication, transforming how doctors, patients, and medical professionals interact. Its immersive technologies facilitate a deeper level of engagement, allowing for more dynamic consultations and more precise educational simulations [27-29].

One of its primary advantages is improved accessibility, as virtual platforms eliminate geographical barriers, allowing patients in remote areas to access high-quality medical expertise that might otherwise be unavailable [30-32]. Enhanced collaboration is another key benefit, as multidisciplinary teams can work together in real-time, irrespective of their physical location, streamlining diagnostics, and treatment planning [33,34].

Virtual consultations also create more engaging patient experiences, as AI-driven support systems can improve patient adherence to treatment plans through interactive education [35-38]. Furthermore, the metaverse is revolutionizing medical education by offering extended reality platforms that provide safe and controlled environments for training future physicians, ensuring higher competency levels before real-world application [39,40].

Cons: A Barrier to Effective Interaction

Despite these advancements, the metaverse presents significant obstacles that may hinder its effectiveness in real-life medical communication [41-43]. One major concern is the loss of human connection in virtual consultations. The digitalization of consultations could lead to reduced emotional engagement, where the subtleties of face-to-face interactions, such as body language and microexpressions, are lost [44,45].

The metaverse may act as a Trojan horse in medical training, offering innovative learning tools while inadvertently weakening essential clinical skills. Over-reliance on virtual environments risks depriving young physicians of the hands-on experience needed for accurate diagnosis, patient rapport, and intuitive medical decision-making. Additionally, technological disparities remain a major concern. The requirement for specialized equipment and stable internet access may widen healthcare inequities, disadvantaging those without access to such resources [46,47].

Data security concerns also pose a significant challenge. The exchange of sensitive patient information in virtual spaces raises ethical concerns about privacy, cybersecurity, and compliance with data protection regulations [48-50]. Another notable issue is cognitive overload. Extended exposure to virtual environments may increase mental fatigue, reducing the effectiveness of virtual consultations and learning sessions. Integrating metaverse technology into clinical practice raises regulatory concerns, including compliance with existing frameworks. Ensuring patient data privacy, ownership, and interoperability in virtual spaces remains a substantial challenge due to the distributed nature of these platforms. Furthermore, informed consent processes may be uniquely influenced by the immersive and novel aspects of metaverse environments. Thus, it is essential to develop consent protocols explicitly addressing the risks, benefits, limitations, and the novelty of these technologies, while honoring patient autonomy, transparency, and trust [51,52].

“Towards a More Human Physician?”

Amid these pros and cons, the most crucial question is not whether the metaverse is merely a tool or a barrier but how it can be leveraged to restore the essence of human-centered medicine. By streamlining administrative tasks and improving efficiency, metaverse-based solutions may allow physicians to dedicate more time to the human aspects of care-offering more profound, meaningful patient interactions. The ability to automate technical processes should ideally create space for doctors to be more present, empathetic, and engaged [53].

 As Eric Topol states in *Deep Medicine*, "The gift of time is the greatest potential reward of AI in medicine. If properly deployed, it can reduce the burden of administrative tasks, allowing doctors to listen, connect, and truly care for their patients" [54]. Yet, as these innovations unfold, a fundamental question remains: Will these new communication pathways ultimately bring physicians closer to their patients, making them more humane, compassionate, and connected? Only time will tell.

## Conclusions

The metaverse is redefining healthcare communication, offering new possibilities for collaboration, education, and patient care. While its potential to enhance accessibility, training, and decision-making is undeniable, challenges such as data security, ethical concerns, and the risk of diminished human connection must not be overlooked. Beyond its technological impact, the true measure of success will lie in how these innovations preserve the human part of medicine, ensuring that digital advancements do not distance, but rather bring physicians and patients closer in meaningful ways. As we step into this new frontier of healthcare, it is important to integrate the advantages of the metaverse responsibly, not as a replacement for human interaction, but as a tool to enrich the compassion and empathy of medical practice.
